# The Psychological Consequences of COVID-19 and Lockdown in the Spanish Population: An Exploratory Sequential Design

**DOI:** 10.3390/ijerph17228578

**Published:** 2020-11-19

**Authors:** María Dolores Hidalgo, Nekane Balluerka, Arantxa Gorostiaga, José Pedro Espada, Miguel Ángel Santed, José Luis Padilla, Juana Gómez-Benito

**Affiliations:** 1 Department of Basic Psychology and Methodology, Faculty of Psychology, University of Murcia, 30100 Murcia, Spain; mdhidalg@um.es; 2 Department of Clinical and Health Psychology, and Research Methods, Faculty of Psychology, University of the Basque Country UPV/EHU, 20018 Donostia, Spain; nekane.balluerka@ehu.eus; 3 Department of Health Psychology, Faculty of Social Health Sciences, Miguel Hernández University, 03202 Elche, Spain; jpespada@umh.es; 4 Department of Personality Psychology, Psychological Assessment and Treatment, Faculty of Psychology, National Distance Education University (UNED), 28040 Madrid, Spain; msanted@psi.uned.es; 5 Department of Methodology for Behavioural Science, Faculty of Psychology, University of Granada, 18071 Granada, Spain; jpadilla@ugr.es; 6 Department of Social Psychology and Quantitative Psychology, Faculty of Psychology, University of Barcelona, 08035 Barcelona, Spain; juanagomez@ub.edu; 7 Group on Measurement Invariance and Analysis of Change (GEIMAC), Institute of Neuroscience, University of Barcelona, 08035 Barcelona, Spain

**Keywords:** COVID-19, lockdown, psychological distress, mixed methods

## Abstract

The objectives of this study were to analyze the psychological impact of the COVID-19 pandemic and the lockdown in the Spanish population and to identify what population profiles were most affected. The study used a sequential exploratory design. In the qualitative phase, 40 participants were recruited based on theoretically relevant criteria and the saturation of the information provided by the interviews. In the quantitative phase, a large representative sample was applied. The universe considered was the adult population of Spain. A total of 6789 surveys were conducted. Both the analysis of the narratives of the interviews and the responses to the panel survey showed relevant changes in attitudes and mood swings compared to the period prior to lockdown. These changes include dysphoric moods (i.e., experiences of distress such as sadness/depression, anxiety, rage, feeling of unreality, worry, etc.) and also some euphoric moods (i.e., feelings of well-being, happiness, etc.). A higher number of women were affected than men and a greater increase was observed in younger people. The findings of the study may serve as a basis for detecting needs and providing psychological support, as the symptoms detected as the most common are key for the processes of screening at-risk individuals.

## 1. Introduction

The crisis resulting from the COVID-19 epidemic has caused extraordinary changes in the population worldwide. Stressful events that pose a threat to survival have significant psychological repercussions. The emotional impact of the fear of contagion and the fear of the illness and/or death of loved ones has been added to the stress resulting from the social distancing and lockdown measures for containing the pandemic. In general, the quarantines due to the pandemic have been associated with acute stress and adaptation stress, symptoms of posttraumatic stress, posttraumatic stress, fear, sadness, nervousness, guilt, confusion, and anger [1]. During lockdown, relational functioning among family, community, and peers is inhibited or interrupted, which can increase vulnerability to adverse reactions from a psychological point of view. Similarly, the incidence of posttraumatic stress disorder (PTSD) after the SARS pandemic in Canada was found to be similar to that of natural disasters and terrorism [2,3].

However, the worldwide lockdown experienced in the first half of 2020 was unprecedented, and has affected millions of inhabitants around the world. We must know what the psychological consequences of COVID-19 and the lockdown are to understand how people have coped with quarantine and what the long-term psychological effects of this situation may be, in order to have a chance to prevent them. Studies conducted in various countries and geographic regions have evidenced the psychological and mental health impact of lockdown [4,5,6,7,8]. These studies have observed an increase in symptoms of depression, anxiety, stress, fear, problems sleeping, and PTSD, although it may not be possible to generalize their results to the population due to the sample size involved or the sample selection procedure used (incidental or snowball recruitment). Several authors have indicated the importance of rigorously analyzing the psychological effects of the COVID-19 pandemic on the population [9,10]. In this sense, cross-sectional population studies such as Qiu et al. [11], Xin et al. [12], and Zhang et al. [13] in China, Lauri Korajlija and Jokic-Begic [14] in Croatia, Alonzi et al. [15] in Canada, Gualano et al. [16] in Italy, and Fitzpatrick et al. [17] in the United States have confirmed the effects of lockdown and COVID-19 on psychological distress.

Spain is one of the countries with the highest rates of people affected and deceased in relation to its number of inhabitants. The mandatory at-home lockdown for the entire Spanish population led to significant habit changes that affect individual well-being [18]. Although some studies with Spanish samples have been published (i.e., [19,20]), studies are needed at the population level, that would be representative enough to delving deeper into the factors related to the psychological impact of COVID-19 and generalizing the findings to the population.

The urgent need for representative data on the psychological effects of COVID-19, as well as the complexity of identifying and evaluating the resulting psychological consequences, is a methodological challenge that requires a complex approach. This study seeks to respond to these challenges through a mixed method study that combines qualitative and quantitative methodology. Mixed methodology has received growing attention in recent decades, as it demonstrates the benefits of combining qualitative and quantitative methods to approach complex phenomena [21,22]. Furthermore, as stated in the review by Brooks et al. [1] of studies on the psychological impact of quarantine, three [23,24,25] of the 24 analyzed studies used a mixed methods design, although all those studies collected data retrospectively and used a cross-sectional survey method in the first phase, later applying a qualitative method (focus groups, semi structured interview, etc.), limiting the scope of the results.

The primary objective of this study was to analyze the psychological impact of the COVID-19 pandemic and of lockdown and its relationship to sociodemographic variables. In addition to learning what the most common symptoms were, we wanted to discover in which population profiles each of the discovered impacts occurred most often.

In order to contribute to filling the detected gaps, this study used a mixed method research design for the first time in this context, using a sequential exploratory design that involved the use of both in-depth interviews and a survey questionnaire, which offers a more comprehensive multimethod view than a design based exclusively on questionnaires. The two methodologies were used sequentially. The experiences of the psychological and emotional consequences of COVID-19 were explored through in-depth interviews conducted during lockdown, and in the second phase of the research, the information obtained from the interviews was used both to identify the profiles for the population study and to develop the questionnaire that was applied by surveying a large representative sample, examining the psychological impact of the epidemic in the last phase of lockdown in the Spanish population.

## 2. Materials and Methods

**Figure 1:**
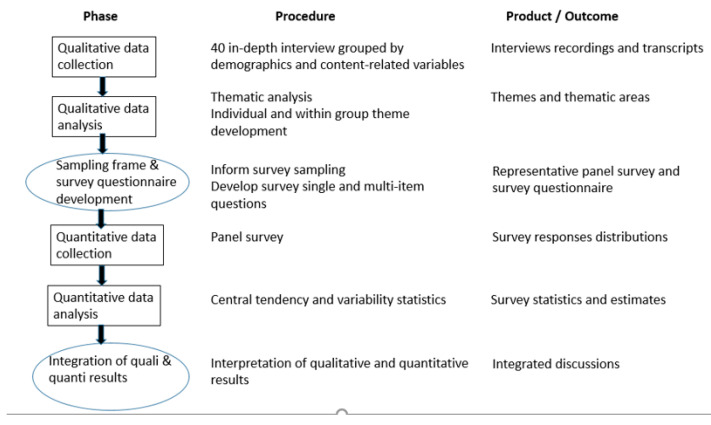
Virtual display of exploratory sequential design.

Figure 1 illustrates how the different phases of the exploratory sequential design were carried out. In terms of the method, we used 2 approaches for integration: “connecting” and “building” [26]. The analysis of in-depth interviews helped to identify the demographic profile of the future participants in the survey study, thus “connecting” the qualitative and quantitative study through the sampling design. At the same time, the subject areas and subjects identified in the analysis of the interviews were used to propose the dimensions and indicators of the questionnaire used in the survey study.

### 2.1. Qualitative Phase

#### 2.1.1. Participants

In total, 40 people were recruited. These participants were chosen considering relevant sociodemographic and substantive characteristics. In addition to considering gender (21 women and 19 men) and age (12 individuals between age 18 and 35, 18 between 35 and 55, and 10 over 55), the individuals resided in different geographic regions, had different work situations (10 temporarily unemployed people, 8 people working remotely), family and living situations, and relationship to COVID-19 (22 people not affected by COVID-19 nor those around them, 8 with people around them diagnosed with the illness). Interviews were conducted until saturation was reached. In this study, saturation was defined as the point when information collected during interviews reveals no additional themes.

The interviewees were chosen from 167 people who accepted; there were only 6 refusals because of the discomfort that talking about COVID-19 could cause the person. The individuals contacted were informed of the study objective and characteristics; they were also given the confidentiality guarantees established in the European General Data Protection Regulation (EU 2016/679), and informed of the approval of the study by the Ethics Committee on Research with Human Beings of the University of the Basque Country (Ethical number M10_2020_076).

#### 2.1.2. Interview Protocol

To develop the protocol, we reviewed the scientific literature on the effects of COVID-19, instruments for evaluating the psychological consequences of the pandemic, and we had the experience of the research team on psychological impact in traumatic situations.

#### 2.1.3. Data Collection and Analysis

The interviews were conducted by 4 interviewers with degrees/doctorates in psychology with experience in conducting in-depth interviews. The interviews were conducted by phone in the second week of April 2020, and were recorded with the consent of the interviewees. The interviewers were trained on the interview protocol, probing techniques and how to manage cases in which the interviewee was referred for psychological care due to showing indicators of clinically significant distress. The duration of the interviews ranged from 25 to 97 min, with an average duration of 53 min.

The research team carried out the analysis of the interviews, guided by the principles of grounded theory [27]. Without using a detailed theoretical outline, the team created a code system to organize and manage the interview conversation. The recordings of the interviews were processed using an Excel sheet in which the code system had been recorded. The analysis was done in 3 phases to progressively break down the data following the comparative method [27]. The analysis paid particular attention to the examination of the interpretative patterns identified through subgroups of people defined by their profiles or experiences of the psychological consequences of COVID-19 and of lockdown. A process consisting of various rounds of review was completed on the first analyses conducted by each analyst on the team to resolve disagreements in processing the information. Any discrepancies were resolved by consensus.

### 2.2. Quantitative Phase

#### 2.2.1. Setting

The survey data collection occurred between 22 and 26 April 2020. During this period, the state of emergency initially declared by Royal Decree 463/2020 (14 March) was extended. In terms of the pandemic in Spain at this time, out of a population of 47,329,981 inhabitants, there was a reported total of 202,990 cases of COVID-19 confirmed by PCR, 22,524 deaths, 92,355 recoveries and positive antibody test results in 16,774 individuals, being the European country with the highest number of confirmed cases (Update no. 85. coronavirus disease (COVID-19)).

#### 2.2.2. Participants

The context considered in the survey study was the adult population (18 years or over) of Spain. For sampling, the key sociodemographic variables of the population were considered as quotas (gender, socioeconomic level, and age), supported by the results of the qualitative study. From the initial sampling, 6789 surveys were ultimately conducted, of which 52.2% were women. Distribution by age was 26.55% for the age range from 18 to 34, 52.1% for the age range from 35 to 60, and 21.4% for the age range over 60. Further, the distribution of participants for the variable socioeconomic level was 36.9% low, 39.8% medium, and 23.4% high. Sampling error was 1.86 with respect to the expanded sample—in other words, the adult population of Spain (34,506,804 people).

#### 2.2.3. Data Collection

The method used to collect information was online surveys, using Netquest Panel Online, composed of the Spanish population over 14 years of age. The maintenance of the panel is as indicated in the ISO 26,362 standard. Of the 16,205 invitations extended, 10,059 were accepted, representing a rate of 62%. Of the 10,059 participants, the following were excluded: 271 due to various quality filters, 42 for follow-up care, 45 because the quota for the autonomous community had already been met, 2297 because they accessed to the survey after the deadline and 615 due to being incomplete.

The online questionnaire was developed based on the thematic areas identified in the narratives of the in-depth interviews, the international scientific literature review on the psychological effects of COVID-19, and the review of instruments for evaluating the psychological effects of COVID-19. The general survey question was: During lockdown, compared to your life before lockdown, how much do you think you have changed with regard to the following aspects? The following response scale was used: 1 = Has decreased a lot; 2 = Has decreased a little; 3 = Has stayed the same; 4 = Has increased a little; 5 = Has increased a lot. The online questionnaire included several quality controls to prevent automated responses and acquiescence response bias. Item quality controls were stablished, thus when a small number of people had responded (5% of the sample), item statements were reviewed. The average response time for the survey was 14.1 min (SD = 6.8) and the average time per page was 0.37 min (SD = 0.18).

#### 2.2.4. Data Analysis

Percentages of change and Chi-square statistical tests for contingency tables were calculated for each of the psychological variables by gender, age, and socioeconomic level.

## 3. Results

The results are presented by merging the findings of the in-depth interviews with the results of the survey study. The report on the results is structured by three sociodemographic variables: gender, age, and socioeconomic level, to make it more useful for reaching conclusions on the psychological effects of COVID-19 in Spain.

Based on the analysis of the discourses with interviewed individuals, we identified changes in their attitude and mood swings compared to their life prior to lockdown as well as changes during lockdown. These changes mainly include dysphoric moods (i.e., experiences of distress such as sadness/depression, anxiety, anger, feeling of unreality, worry, etc.) and also some euphoric moods (i.e., feelings of well-being, happiness, etc.).

This general pattern of findings in the interviews converges with the primary results obtained in the survey study. Table 1 presents the primary themes that emerged from the analysis of the interviews, as well as their correspondence with the survey study results.

### 3.1. Psychological Changes by Gender

The in-depth interviews already pointed out gender differences in the experience of psychological changes as a consequence of COVID-19. For example, some emotional changes seem to be associated with the role of being a mother. One interviewee reported that everything has changed for her, both in chores and in strains: the children’s homework, more cleaning, stress, feeling overwhelmed, psychological exhaustion, uncertainty...

“This is hard and here at home there have been days when one of my children has been truly down, despairing, and the tears start to fall.”

(IN19_H_33)

Another interviewee reports:

“I’m more nervous, everything is mounting up, the children, the house, that my husband is out there. I’m scared.”

(IN23_M_42)

Table 2 shows survey estimates per gender group for each of the psychological variables. Due to the high sample size, all Chi-square statistical tests were significant (*p* < 0.001). Thus, psychological variables with greater percentage differences between females and males are discussed. Women, in a higher percentage than men, reported a greater increase in indicators of psychological distress, difficulty concentrating, feelings of depression, feelings of guilt, mood swings, problems sleeping, levels of irritation, panic attacks, tendency to not want to think, feelings of unreality, and physical symptoms without a clear relationship to the illness. Furthermore, a higher percentage of women than men indicated that their fear of losing loved ones had greatly increased and that their feelings of confidence, peace and vitality/energy had decreased.

### 3.2. Psychological Changes by Age

All Chi-square statistical tests were significant (*p* < 0.001). Psychological variables with greater percentage differences by age are discussed. Table 3 shows differences in younger people, compared to the other age groups, regarding a greater increase in general distress, difficulty concentrating, uncertainty, panic attacks, tendency not to think, feelings of depression, feelings of guilt, thoughts of self-harm, feelings of loneliness, irritation or anger, mood swings, problems with sexual intercourse, problems sleeping, and psychosomatic symptoms. Participants in this age group also reported a greater decrease in their ability to make decisions, in their feelings of peace and calm, and in their feelings of vitality.

The narratives of the youngest participants evidenced adverse effects of overwhelm and distress, for example:

“I feel overwhelmed.”

(IN01_M_22)

“Our exams have been suspended, they are going to be delayed and they haven’t told us when they will be and it feels uncertain since you don’t really know what is going to happen.”

(IN18_F_30)

In contrast, people over age 60 presented a greater increase in concern for suffering from or contracting a serious disease, compared to other age groups. The narratives of this age group evidence this also:

“I was very afraid; my children lost their father in November and I don’t think they are ready to lose their mother.”

(IN35_F_60)

Furthermore, in this same age group, there was a greater increase in willingness to help other people than in other groups. This is also reflected in the interviews:

“The best... also the solidarity that everyone has demonstrated. Thinking about others and being more human in general, in our life, that remains and will remain, for those of us who have lived through this experience, it will remain...”

(IN36_M_62)

### 3.3. Psychological Changes by Socioeconomic Level

Although all Chi-square statistical tests were significant (*p* < 0.001), as shown in Table 4, the patterns of percentages of change in the psychological variables analyzed were similar regardless of the socioeconomic level of the participants in the survey study. However, survey respondents in the highest socioeconomic levels indicated a higher percentage of a slight or high increase in willingness to help others than those in the lower levels. In contrast, a greater percentage of people from medium and low socioeconomic levels indicated an increase in feeling depressed, pessimistic, or hopeless, panic attacks, thoughts of self-harm, as well as a greater reduction in feelings of vitality and energy than people from the highest socioeconomic level. The differences indicated were not large.

In interviews with people who had financial problems, some of these dysphoric feelings were mentioned:

“I am struggling a little at night, I wake up and I get a little panicked because I can’t see an easy solution to this.”

(IN17_F_52)

“The work situation comes to mind, the uncertainty, when will it resume, whether it’s going to be the same...”

(IN26_M_35)

In contrast, some of the people who expressed that they had not been financially affected expressed worry for other people who had it worse and their willingness to help:

“I’m worried that people will suffer.”

(IN20_F_56)

“The volunteer work I do is delivering food: we go to the location they have, there are shopping trolleys, we fill them with purchases, people come and get the food.”

(IN40_M_21).

## 4. Discussion

The primary objective of this study was to analyze the psychological impact of the COVID-19 pandemic and of lockdown in the Spanish population and to identify what population profiles were most affected. For this purpose, we used a mixed research methodology for the first time in this context to obtain a more comprehensive view, combining the advantages of qualitative and quantitative methodologies, where the data collection was done while the population was in lockdown. Both the analysis of the narratives of the interviews and the responses to the panel survey showed relevant changes in attitudes and mood swings compared to the period prior to lockdown. Changes in sleep were one of the indicators of well-being that were most impacted, which is in line with the observations from studies with other populations [6,28].

With regard to population profiles, a higher number of women were affected than men. Most women reported having experienced mood swings, compared to a much lower rate in men. Feelings of depression were also more frequent in women. Furthermore, women more frequently reported having feelings of guilt compared to men. This pattern was also observed in specific cognitive symptoms, such as difficulty concentrating, more present in women, with twice as many participants experiencing a decline. This tendency was maintained in other symptoms related to other mood symptoms, noting that women had higher levels of irritability. In the area of activation and anxiety, close to double the rate of anxiety attacks was observed in women than men. These data are consistent with the analysis of the in-depth interviews, where the narratives were in line with this. Some emotional changes seem to be associated with the role of being a mother. As in previous studies in the Spanish population [20], women have suffered a greater degree of negative psychological impact. A similar pattern was observed in the international population [6,16,29]. The reason could be related to the different way in which gender roles affect men and women, including in the context of the pandemic, which routinely involves greater responsibility as family caregivers. Therefore, the interruption of schooling may have further increased their level of stress [30].

In terms of age, a greater increase was observed in younger people in general distress, difficulty concentrating, levels of uncertainty, anxiety attacks, feelings of depression, feelings of guilt, loneliness, irritation or anger, mood swings, problems with sexual intercourse, problems sleeping, and psychosomatic symptoms. Young people also reported a greater decrease in their ability to make decisions, in their feelings of peace and calm and in their feelings of vitality, in keeping with other Spanish studies [18]. The reason for this difference may be due to the different levels of demands and personal resources. Although it is true that the financial impact due to job loss more directly affects adults, the temporary measures may have also helped to reduce uncertainty about the future. However, the changes in academic activity in the youngest participants (e.g., online schooling), seem to have affected their emotional stability to a greater degree. Another possible explanation is related to personal coping resources, which are less developed in the juvenile population, therefore making subjects feel less confident in facing this crisis [31]. The content of worries varies by ages, noting that people over age 60 presented a greater increase in worry about suffering from or contracting a serious disease compared to other age groups. Older age has been identified in previous studies as a factor for vulnerability to negative impact in traumatic situations [32]. In the sample from this study, the age range of older individuals was sufficiently represented, therefore these data complement the recommendations to explore the negative impact of COVID-19 in the elderly population [18].

With regard to the degree of psychological impact based on socioeconomic level, similarly to other studies with Spanish participants [20], we found that people with higher incomes tended to show less psychological impact than those from a lower socioeconomic level. Despite this, the changes in the psychological variables analyzed were similar regardless of the socioeconomic level of the participants in the survey study. In the higher socioeconomic levels, a tendency was observed towards the willingness to help others, irritation and anger, mood swings, and general psychological distress. People with medium and low socioeconomic levels demonstrated a greater degree of increase in feelings of depression, anxiety attacks and suicidal ideation. They also showed a greater reduction in feelings of vitality and energy compared to people with a higher socioeconomic level. The role of socioeconomic level as a protective factor was confirmed in studies such as Cao et al. [4], who observed that living in urban areas and having financial stability acted as protective factors against generalized anxiety in the Chinese population.

This study has limitations that should be pointed out; primarily in the design, which, given its cross-sectional nature, does not allow for establishing causal or predictive relationships based on the data found. In addition, the data collection procedure was based on subjective self-reported measures based on subjects’ assessment of their own condition. The data were collected during the final phase of at-home lockdown; therefore, the results provide data relative to that moment in time. One possible limitation with respect to interview analysis was that a double coding in order to establish intercoder reliability was not conducted. However, a process consisting of several rounds of review of the preliminary analysis conducted by each team member was carried out, where disagreements were resolved by consensus. Despite this, this study provides solid, novel points as it is the first study using a mixed qualitative and quantitative methodology, which allows for people’s experiences to be transferred to the panel survey. Furthermore, the data come from a large sample collected with international quality criteria to be representative of the Spanish population. The findings of the study may serve as a basis for detecting needs and providing psychological support, since the symptoms detected as the most common are key for the processes of screening at-risk individuals.

## 5. Conclusions

The study provides solid, novel points as it is the first study using a mixed qualitative and quantitative methodology, which allows for people’s experiences to be transferred to the panel survey. Furthermore, the data come from a large sample created with international quality criteria to be representative of the Spanish population.

Both the analysis of the narratives of the interviews and the responses to the panel survey showed relevant changes in attitudes and mood swings compared to the period prior to lockdown. These changes include dysphoric moods and also some euphoric moods. A higher number of women were affected than men and a greater increase of dysphoric moods was observed in younger people.

The findings of the study may serve as a basis for detecting needs and providing psychological support, as the symptoms detected as the most common are key for the processes of screening at-risk individuals.

## Figures and Tables

**Table 1:** Themes, survey questions and survey estimates.

Theme	In-Person Interviews	Survey Question ^a^	Survey
Uncertainty	*“I am lucky that I don’t have family members or friends that have been or are affected. That would change everything, if my mother or my daughters were in the hospital it would be different: more uncertainty, sadness, panic.” (IN11_M_43)*	Uncertainty	In 77.5% of survey respondents, increased slightly (37.8%) or greatly (39.7%)
Symptoms on the depression spectrum	*“The emotion of sadness, of apathy, of saying you don’t even feel like showering or anything... why should I get dressed or comb my hair, they’re things we have to do to keep up a routine, but as the days go it gradually stops.” (IN26_M_25)* *“This is hard and here at home there have been days when one of my children has been truly down, despairing, and the tears start to fall.” (IN19_M_33)*	Feelings of depression, pessimism, or hopelessness	In 43.2% of survey respondents, increased slightly (33.4%) or greatly (9.8%)
Symptoms of anxiety, or reactions of anxiety, and feelings of loneliness	*“Work gives me anxiety and being at home I can’t manage it and it’s with me all day. Once I get started it’s OK... when I finish working, I’m exhausted. My anxiety is worse, it was bad before, the anxiety, but now I feel very alone.” (IN02_F_35)* *“…, but now at home, with the anxiety... I can’t stop myself from snacking on a handful of crisps or chocolate.” (IN02_F_35)*	Panic and anxiety attacks Feelings of loneliness	In 35.1% of survey respondents, panic increased slightly (27.1%) or greatly (8%) In 34.6% of survey respondents, feelings of loneliness increased slightly (24.9%) or greatly (9.7%)
Overwhelm/psychological distress	*“I am starting to feel psychologically exhausted” (IN06_ M_41)* *“These are very difficult times. I am losing a lot of people; 4 family members and people close to me. You try to go out, take it in and they phone you and someone else... It’s very difficult. You cry and ask for psychological help.” (IN05_F_44)* *“Psychologically it’s going to affect us all, not being in contact with anyone, talking face to face, connecting, …” (IN21_M_24)*		In 45.7% of survey respondents, increased slightly (36.2%) or greatly (9.5%)
Worry about suffering from a serious disease	*“...I haven’t left my house again, I had a thing in my lung and it scares me, I don’t go out, my husband comes, he comes with the shopping and we clean everything” (IN6_F_41).* *“…, I’m also scared of something happening to me” (IN35_F_60)*	Worry about suffering from or contracting an illness (COVID-19 or other)	In 67.9% of survey respondents, worry about illness increased slightly (39.4%) or greatly (28.5%)
Irritation or anger	*“At the beginning you fight the system a little, you are kind of against everything, and then you start to realize that it’s a very serious matter, that it’s not about you anymore, it’s about everyone else, that you have to stop and keep calm. And you also are seeing the news and you go from rebellion to alarm, fear, not knowing, the uncertainty, the nervousness...” (IN28_F_47)*	Irritation or anger	In 47.4% of survey respondents, increased slightly (37.6%) or greatly (9.8%)
Fear of the death of loved ones	*“Now it’s my mother who is in hospital and I’m afraid of how it’s going to progress. I am worried, I lost my father a week ago and I’m afraid of losing her, it’s distressing.” (IN25_M_41)* *“I was very afraid; my children lost their father in November and I don’t think they are ready to lose their mother.” (IN35_F_60)*	Fear of losing loved ones	75.5% of survey respondents increased slightly (40.4%) or greatly (35.1%)
Vitality-Energy	*“In the mornings I don’t have energy and it’s hard to start working. Then during the day, since I’m distracted, I don’t think about things and at night when everyone goes to sleep, I feel like I get a little worse again, I think more about the situation we’re in, I’ve been a roller coaster. These days I’ve been telling my friends and family members that I love them a lot.” (IN01_M_22)*	Feelings of vitality and energy	In 48.6% of survey respondents, these feelings decreased slightly (37.6%) or greatly (11%)
Disconnecting	*“We are quite the homebodies, unconsciously because we are working and living in the same space, in the end, the work problems are there all the time, you don’t disconnect. Nervousness or anxiety is more due to work, that I can’t disconnect, more than due to being at home. Before I could discharge more energy and now I can’t.” (IN02_F_35)* *“When I was studying I could go out and disconnect, but now I can’t.” (IN26_M_35)*	Ease of disconnecting from worries	In 34.3% of survey respondents, capacity to disconnect decreased slightly (24.5%) or greatly (9.8%)
Perception of control, being positive, relaxation	*“When I found out I was working, and when I got home it hit me. I thought, ‘what do I do?’ The first days are hard due to your work, not continuing, worrying, and then you say, ‘I have to overcome this and try to be positive,’ for everyone around you, as well.” (IN28_F_47)*	Feelings of confidence and optimism Feelings of peace, calm, relaxation	In 43.5% of survey respondents, feelings of optimism decreased slightly (35%) or greatly (8.5%). These feelings increased in 14.3% of survey respondents. In 43.9% of survey respondents, feelings of relaxation decreased slightly (33.1%) or greatly (10.8%). These feelings increased in 12.3% of survey respondents.
Problems and/or changes in sleep	*“I have had to take sleeping pills” (IN14_F_55)*	Problems sleeping	In 52.8% of survey respondents, this problem increased slightly (32.7%) or greatly (20.1%)
Suicidal thoughts Suicidal ideation	*“A thousand things go through your mind, I think everything is over, nothing will be the same again; sometimes I think I don’t want to go on in this life, but then I think, no, I can’t talk that way, I have my daughters, my husband and my parents.” (IN05_F_44)*	Thoughts of self-harm	In 4.5% of survey respondents, this type of ideation increased slightly (3.5%) or greatly (1%)
Unreality	*“The first days it felt unreal, something is happening that has never happened and it just doesn’t seem normal” (IN18_F_30)*	Feelings of unreality, that things are not real	In 42.7% of survey respondents, these feelings increased slightly (31.0%) or greatly (11.7%)
Concentration	*“Because I’m not studying anything this month, I can’t concentrate, I’m sure my exam will be postponed until next year, I don’t know.” (IN6_F_41)* *“it’s hard to concentrate at home” (IN40_M_21)*	Difficulty concentrating	In 41.2% of survey respondents, difficulty focusing increased slightly (29.1%) or greatly (12.1%)
Not thinking	*“So it’s better to stop thinking about it and live day to day” (IN23_F_42)* *“...I try not to watch the news, not to think about it, to be at peace with the children.” (IN6_F_41)*	Tendency to not want to think and to not talk about problems	In 31.9% of survey respondents, increased slightly (24%) or greatly (7.9%)
Guilt	*“You go out and it feels like you’re committing a crime, that’s what makes me the most angry, even going to get bread or tobacco you feel bad. The TV chips away at your morale.” (IN4_M_62)*	Feelings of guilt	In 13.8% of survey respondents, guilt feelings increased slightly (11.1%) or greatly (2.7%)
Prosocial behavior	*“Next week we’re going to start helping at an organization.” (IN18_F_30)* *“The volunteer work I do is delivering food: we go to the location they have, there are shopping trolleys, we fill them with purchases, people come and get the food.” Also, sometimes I take shopping to my grandmother.” (IN40_M_21)*	Willingness to help others (donations to NGOs, family members, etc.)	In 36.2% of survey respondents, prosocial behavior increased slightly (25.9%) or greatly (10.3%)
Somatic symptoms	*“I’m getting a muscle spasm.” (IN38_F_29)* *“It’s hard for me to breathe, I sigh, I’m close to tears, I’m terrified to start the day…” (IN2_F_35)*	Physical symptoms without a clear relationship to a medical condition	In 31.4% of survey respondents, somatic symptoms increased slightly (24.3%) or greatly (7.1%)
Mood swings	*“I have days where I’m desperate to go out and times when I’m just down.”* *(IN25_M_41)*	Mood swings	In 44.7% of survey respondents, increased slightly (34.2%) or greatly (10.5%).
Coping with problems	*“Friends, family members, who are going to sort of solve the problems you might come to have.” (IN11_M_43)* *“Above all I have needed information, I’ve had to pay a lot of attention.” (IN9_F_50)*	Ability to make decisions and solve problems	In 14.1% of survey respondents, capacity to solve problems decreased slightly or greatly. In 17.9% of survey respondents, it increased slightly or greatly.

^a^ General Survey question: During lockdown, compared to your life before lockdown, how much do you think you have changed with regard to the following aspects?

**Table 2:** Percentages of psychological changes during lockdown by gender.

Psychological Variables	Gender	1	2	3	4	5
General psychological distress	Female	1.7	3.6	42.7	39.7	12.1
	Male	2.3	3.3	54.8	32.6	6.8
Difficulty concentrating	Female	3.1	7.8	42.5	30.4	16.1
	Male	1.8	7.8	54.7	27.8	7.8
Ease of disconnecting from worries	Female	12.6	25.0	38.8	17.0	6.4
	Male	6.9	24.0	47.9	17.0	4.2
Uncertainty	Female	0.9	1.2	17.1	35.2	45.4
	Male	1.2	1.7	22.6	40.5	33.8
Panic attacks	Female	3.2	2.4	49.6	33.2	11.3
	Male	3.4	3.2	67.9	20.6	4.6
Worry about suffering from or contracting a serious disease	Female	1.9	1.2	28.1	38.2	30.2
	Male	1.5	2.8	28.0	40.7	26.7
Tendency to not want to think and to not talk about problems	Female	2.0	5.1	54.8	27.3	9.8
	Male	2.0	6.5	64.7	20.6	6.0
Feelings of depression, pessimism, or hopelessness	Female	2.8	3.6	43.2	37.8	11.9
	Male	2.9	4.0	56.3	28.9	7.6
Feelings of guilt	Female	3.5	3.6	74.8	12.9	4.1
	Male	3.4	4.4	81.5	9.1	1.3
Thoughts of self-harm	Female	9.8	2.2	79.2	3.4	1.3
	Male	8.8	3.0	81.8	3.7	0.7
Fear of losing loved ones	Female	0.5	0.8	18.6	38.9	40.5
	Male	0.8	0.6	26.9	42.0	29.5
Feelings of loneliness	Female	4.2	5.6	51.6	27.0	11.3
	Male	3.2	5.1	60.9	22.7	8.0
Irritation or anger	Female	2.1	3.7	41.6	40.3	12.1
	Male	2.1	5.1	50.7	34.8	7.3
Mood swings	Female	1.5	3.2	43.3	38.5	13.5
	Male	1.4	4.9	56.6	29.8	7.3
Feelings of unreality, that things are not real	Female	2.2	1.2	47.1	32.6	15.4
	Male	2.2	2.4	58.0	29.3	7.9
Problems with sexual intercourse	Female	5.2	6.3	65.7	10.9	7.9
	Male	3.7	7.0	67.6	13.2	7.0
Problems sleeping	Female	1.7	4.2	34.8	33.5	25.8
	Male	1.5	5.0	47.3	31.9	14.2
Ability to make decisions and solve problems	Female	2.9	12.8	65.2	13.3	5.7
	Male	2.1	10.4	70.8	12.1	4.6
Feelings of confidence and optimism	Female	10.5	37.1	38.1	11.3	2.8
	Male	6.4	32.8	46.2	11.6	2.9
Feelings of peace, calm, relaxation	Female	14.1	36.1	33.2	12.3	4.2
	Male	7.5	30.1	45.7	11.5	5.2
Feelings of vitality and energy	Female	14.2	40.0	32.8	10.6	2.4
	Male	7.8	35.2	45.2	8.8	2.9
Willingness to help others	Female	2.5	2.9	53.5	27.3	12.0
	Male	3.5	3.7	59.3	24.2	8.4
Physical symptoms without a clear relationship to a medical condition	Female	2.6	2.8	56.8	28.1	9.1
	Male	1.9	3.1	69.4	20.4	5.0

Note: 1 = Has decreased a lot; 2 = Has decreased a little; 3 = Has stayed the same; 4 = Has increased a little; 5 = Has increased a lot.

**Table 3:** Percentages of psychological changes during lockdown by age.

Psychological Variables	Age	1	2	3	4	5
General psychological distress	18–34	1.1	3.6	41.3	41.6	12.3
	35–60	2.2	3.3	48.1	37.4	8.9
	>60	2.8	3.9	59.0	26.5	7.5
Difficulty concentrating	18–34	2.8	6.0	37.2	34.2	19.7
	35–60	2.6	9.1	47.2	30.6	10.4
	>60	1.7	6.7	65.8	19.1	6.6
Ability to disconnect from concerns	18–34	13.4	27.9	36.4	16.4	5.8
	35–60	9.2	24.7	42.5	17.8	5.7
	>60	6.7	19.7	53.7	15.9	3.9
Uncertainty	18–34	0.7	1.0	18.5	35.0	44.6
	35–60	0.9	1.5	17.9	38.9	40.6
	>60	1.7	2.0	26.3	38.5	31.4
Panic attacks	18–34	1.8	3.6	52.4	30.5	11.4
	35–60	3.5	2.3	59.2	27.5	7.2
	>60	4.5	3.2	64.7	21.6	5.6
Worry about suffering from or contracting a serious disease	18–34	2.6	3.3	32.4	39.6	22.1
	35–60	1.4	1.8	28.3	37.6	30.3
	>60	1.4	0.8	21.8	43.9	31.9
Tendency to not want to think and to not talk about problems	18–34	1.5	6.5	53.9	26.8	10.7
	35–60	1.7	5.3	61.0	24.5	7.0
	>60	3.5	6.1	63.5	19.3	6.8
Feelings of depression, pessimism, or hopelessness	18–34	1.6	3.5	44.6	36.7	13.1
	35–60	2.9	3.8	49.5	33.9	9.4
	>60	4.2	4.0	56.5	28.2	6.8
Feelings of guilt	18–34	2.1	4.1	75.1	13.5	4.7
	35–60	3.4	3.6	77.8	12.1	2.4
	>60	5.1	5.0	82.8	5.4	1.0
Thoughts of self-harm	18–34	7.4	1.6	82.5	3.2	2.0
	35–60	10.1	2.8	79.2	3.9	0.8
	>60	9.7	3.4	81.0	3.2	0.4
Fear of losing loved ones	18–34	0.4	0.9	24.9	41.2	32.2
	35–60	0.6	0.7	22.2	39.2	37.1
	>60	1.1	0.5	21.3	42.6	34.1
Feelings of loneliness	18–34	2.7	5.7	49.1	29.9	12.5
	35–60	4.8	5.4	58.1	22.5	9.0
	>60	2.0	5.0	60.1	24.7	7.9
Irritation or anger	18–34	1.3	4.0	35.9	44.4	14.3
	35–60	2.4	4.3	46.2	38.7	8.3
	>60	2.5	4.8	58.3	26.2	7.7
Mood swings	18–34	1.0	2.7	40.1	39.8	16.3
	35–60	1.2	4.5	49.0	36.0	9.3
	>60	2.5	4.5	64.0	22.8	6.0
Feelings of unreality, that things are not real	18–34	1.7	1.7	52.1	29.7	13.2
	35–60	2.4	1.4	51.2	33.5	10.7
	>60	2.1	2.9	56.0	26.2	12.4
Problems with sexual intercourse	18–34	5.1	6.5	60.4	14.9	10.1
	35–60	4.6	6.9	66.4	12.6	7.6
	>60	3.3	6.3	75.0	6.9	3.9
Problems sleeping	18–34	1.6	4.8	32.6	31.1	29.9
	35–60	1.6	4.5	40.7	34.1	19.0
	>60	1.8	4.4	52.0	31.3	10.5
Ability to make decisions and solve problems	18–34	3.4	16.6	59.3	13.4	7.2
	35–60	2.3	10.4	68.6	13.9	4.7
	>60	2.3	8.3	77.0	8.7	3.9
Feelings of confidence and optimism	18–34	9.0	34.3	40.7	13.2	2.7
	35–60	8.3	36.3	41.2	11.4	2.7
	>60	8.3	32.7	45.9	9.5	3.4
Feelings of peace, calm, relaxation	18–34	13.0	35.2	33.4	13.4	4.9
	35–60	11.4	33.7	39.1	11.1	4.6
	>60	6.7	29.3	47.5	12.0	4.6
Feelings of vitality and energy	18–34	15.4	38.4	31.3	12.3	2.4
	35–60	10.4	40.5	37.7	8.9	2.5
	>60	7.2	29.7	51.4	8.4	3.2
Willingness to help others	18–34	2.9	3.1	53.6	28.5	10.7
	35–60	3.4	3.7	58.8	23.4	9.2
	>60	2.0	2.5	53.8	28.3	12.4
Physical symptoms without a clear relationship to a medical condition	18–34	2.0	2.3	58.7	28.4	8.4
	35–60	2.2	3.5	61.8	25.0	7.0
	>60	2.7	2.5	71.3	17.4	5.7

Note: 1 = Has decreased a lot; 2 = Has decreased a little; 3 = Has stayed the same; 4 = Has increased a little; 5 = Has increased a lot.

**Table 4:** Percentages of psychological changes during lockdown by socioeconomic level (SEL).

Psychological Variables	SEL	1	2	3	4	5
General psychological distress	High	1.8	3.8	49.5	35.6	9.3
	Medium	2.2	3.3	48.1	36.7	9.4
	Low	1.9	3.4	47.9	36.4	10.0
Difficulty concentrating	High	2.6	6.7	46.4	32.3	12.0
	Medium	2.3	8.2	50.6	27.0	11.9
	Low	2.5	9.0	48.4	27.5	12.6
Ability to disconnect from concerns	High	11.3	25.0	40.7	18.5	4.4
	Medium	9.3	25.4	42.9	16.9	5.5
	Low	8.1	21.8	48.5	14.6	6.6
Uncertainty	High	1.1	1.2	18.9	37.0	41.7
	Medium	0.6	1.6	19.6	40.0	38.1
	Low	1.6	1.7	22.0	35.0	39.1
Panic attacks	High	3.2	2.9	61.5	23.9	8.3
	Medium	3.2	2.6	57.1	29.9	7.0
	Low	3.4	3.1	56.0	27.6	9.3
Worry about suffering from or contracting a serious disease	High	1.5	2.5	29.1	39.4	27.4
	Medium	1.8	1.5	26.8	39.6	29.9
	Low	2.1	2.0	28.2	39.3	27.7
Tendency to not want to think and to not talk about problems	High	2.3	6.3	58.6	24.5	7.8
	Medium	1.6	5.6	60.8	23.8	7.4
	Low	2.3	5.3	59.5	23.4	9.2
Feelings of depression, pessimism, or hopelessness	High	2.6	2.9	52.7	32.1	9.4
	Medium	3.3	4.1	47.7	35.8	8.8
	Low	2.6	4.8	47.8	31.6	12.4
Feelings of guilt	High	2.8	5.2	76.8	12.2	2.8
	Medium	3.9	3.5	79.3	9.9	2.6
	Low	3.8	2.7	78.5	11.3	2.8
Thoughts of self-harm	High	8.8	2.8	83.1	2.4	0.9
	Medium	9.2	2.3	79.9	4.4	0.9
	Low	10.4	2.8	76.6	4.1	1.6
Fear of losing loved ones	High	0.3	0.3	23.5	42.4	33.3
	Medium	0.9	0.8	21.1	40.2	36.6
	Low	0.7	1.2	24.2	37.2	36.0
Feelings of loneliness	High	3.9	6.6	57.5	23.6	8.4
	Medium	3.8	4.7	56.3	26.1	8.7
	Low	3.1	4.3	53.4	25.0	13.9
Irritation or anger	High	2.2	3.8	42.9	40.3	10.6
	Medium	1.9	5.0	48.0	36.7	8.4
	Low	2.4	4.1	48.2	34.3	10.8
Mood swings	High	1.5	2.7	49.1	35.9	10.8
	Medium	1.2	5.0	49.7	34.2	9.9
	Low	1.6	4.7	51.3	31.3	10.9
Feelings of unreality, that things are not real	High	2.0	1.5	50.8	32.0	12.6
	Medium	2.3	1.9	53.5	30.4	11.1
	Low	2.4	2.1	53.5	30.1	11.1
Problems with sexual intercourse	High	4.7	7.2	64.7	13.7	7.3
	Medium	3.7	7.1	68.1	11.4	7.6
	Low	5.4	5.0	67.6	9.8	7.5
Problems sleeping	High	1.6	4.5	39.9	33.9	20.1
	Medium	1.7	5.0	40.7	33.2	19.4
	Low	1.5	4.0	43.3	29.7	21.5
Ability to make decisions and solve problems	High	2.6	12.3	66.3	12.3	6.4
	Medium	2.1	11.2	69.6	12.5	4.5
	Low	3.2	11.1	67.8	13.6	4.1
Feelings of confidence and optimism	High	8.9	35.4	40.4	12.7	2.4
	Medium	7.1	36.5	43.9	10.0	2.6
	Low	10.3	31.7	41.7	11.9	4.2
Feelings of peace, calm, relaxation	High	10.6	33.0	39.2	11.9	5.3
	Medium	11.2	33.7	39.3	11.9	3.8
	Low	10.6	32.5	39.6	12.0	5.2
Feelings of vitality and energy	High	12.2	35.5	38.7	11.1	2.4
	Medium	10.3	39.5	39.6	7.6	3.0
	Low	10.5	38.2	37.9	10.8	2.3
Willingness to help others	High	2.3	3.1	53.9	28.4	11.6
	Medium	2.6	3.3	57.5	25.5	9.7
	Low	4.7	3.6	58.7	21.5	8.9
Physical symptoms without a clear relationship to a medical condition	High	1.7	4.0	64.0	23.4	6.7
	Medium	2.8	2.2	62.5	25.0	7.0
	Low	2.3	2.6	62.1	24.6	8.0

Note: 1 = Has decreased a lot; 2 = Has decreased a little; 3 = Has stayed the same; 4 = Has increased a little; 5 = Has increased a lot.
